# EGG CONSUMPTION: TRENDS OVER TIME, PATTERNS ACROSS THE LIFESPAN, AND PREDICTORS OF INTAKE

**DOI:** 10.1093/geroni/igad104.3296

**Published:** 2023-12-21

**Authors:** Donna Kritz-Silverstein, Ricki Bettencourt

**Affiliations:** University of California San Diego, San Diego, California, United States; University of California San Diego, San Diego, California, United States

## Abstract

Eggs are an excellent nutritional source. However, the historical association of dietary cholesterol with serum cholesterol and thus, cardiovascular disease, along with restrictive dietary guidelines may have served as a barrier to egg consumption. This study examines trends over time, and predictors and patterns of egg consumption in older individuals followed for over 45 years. Participants were 6326 men and women enrolled in the Rancho Bernardo Study in 1972-74 when they were asked about the number of eggs consumed/week. Subsequent egg intake information was collected with food frequency questionnaires from subsamples attending clinic visits in 1988-91 (N=1627) and 1992-96 (N=1385), and with the original question on a 2021 mailed survey (N=710). Mean(□SD) eggs consumed was 3.6□3.0 in 1972-74, 1.8□2.1 in 1988-91, 1.8□2.2 in 1992-96, and 3.4□3.5 in 2021. Comparisons within 5-year categories of baseline age (< 20, 20-24, 25-29, 30-34, 35-39, >40) showed no differences in egg intake between 1972-74 and when older in 2021. Men consumed more eggs than women at all timepoints (p’s< 0.0001). Those with higher education (p< 0.0001) and diabetes (p=0.0009) consumed more eggs; those with high cholesterol or taking cholesterol lowering medication consumed fewer eggs in 1972-74 (p’s< 0.0001), but there were no differences by these factors by 2021. Egg consumption appears responsive to dietary guidelines, decreasing over time then increasing by 2021 to levels similar to the early 1970s. Among those young and middle-aged at baseline, egg intake patterns were similar 45 years later, when older. Cholesterol and cholesterol-lowering medication use are no longer factors in egg intake.

